# Semaglutide vs Endoscopic Sleeve Gastroplasty for Weight Loss

**DOI:** 10.1001/jamanetworkopen.2024.6221

**Published:** 2024-04-12

**Authors:** Muhammad Haseeb, Jagpreet Chhatwal, Jade Xiao, Pichamol Jirapinyo, Christopher C. Thompson

**Affiliations:** 1 Division of Gastroenterology, Hepatology and Endoscopy, Brigham and Women’s Hospital, Boston, Massachusetts; 2 Department of Gastroenterology, Hepatology and Nutrition, University of Pittsburgh Medical Center, Pittsburgh, Pennsylvania; 3 Institute for Technology Assessment, Massachusetts General Hospital, Boston; 4 Georgia Institute of Technology, Atlanta

## Abstract

**Importance:**

Obesity is a disease with a large socioeconomic burden. Endoscopic sleeve gastroplasty (ESG) is a minimally invasive endoscopic bariatric procedure with wide global adoption. More recently, new weight-loss medications, such as glucagon-like peptide-1 receptor agonists (eg, semaglutide), have attracted increased attention due to their efficacy. However, their cost-effectiveness over an extended period compared with ESG is a critical gap that needs to be better explored for informed health care decision-making.

**Objective:**

To assess the cost-effectiveness of semaglutide compared with ESG over 5 years for individuals with class II obesity.

**Design, Setting, and Participants:**

This economic evaluation study, conducted from September 1, 2022, to May 31, 2023, used a Markov cohort model to compare ESG and semaglutide, with a no-treatment baseline strategy. The study comprised adult patients in the US health care system with class II obesity (body mass index [BMI] of 35-39.9). The base case was a 45-year-old patient with class II obesity (BMI of 37). Patients undergoing ESG were subjected to risks of perioperative mortality and adverse events with resultant costs and decrement in quality of life.

**Interventions:**

Strategies included treatment with semaglutide and ESG.

**Main Outcomes and Measures:**

Costs (2022 US dollars), quality-adjusted life-years (QALYs), and incremental cost-effectiveness ratio (ICER) with a willingness-to-pay threshold of $100 000/QALY. A 5-year time horizon with a cycle length of 1 month with a 3% discount rate was used. Probabilities, costs, and quality-of-life estimates of the model were derived from published literature. One-way, 2-way, and probabilistic sensitivity analyses were also performed.

**Results:**

The model found that ESG was more cost-effective than semaglutide over a 5-year time horizon, with an ICER of –$595 532/QALY. Endoscopic sleeve gastroplasty added 0.06 QALYs and reduced total cost by $33 583 relative to semaglutide. The results remained robust on 1-way and probabilistic sensitivity analyses. Endoscopic sleeve gastroplasty sustained greater weight loss over 5 years vs semaglutide (BMI of 31.7 vs 33.0). To achieve nondominance, the annual price of semaglutide, currently $13 618, would need to be $3591.

**Conclusions and Relevance:**

This study suggests that ESG is cost saving compared with semaglutide in the treatment of class II obesity. On price threshold analyses, a 3-fold decrease in the price of semaglutide is needed to achieve nondominance.

## Key Points

**Question:**

What is the cost-effectiveness viability of semaglutide compared with endoscopic sleeve gastroplasty (ESG) over 5 years for individuals with class II obesity?

**Findings:**

In this economic evaluation study using a Markov cohort model analysis, ESG was found to be a cost-effective strategy, offering greater weight loss and cost savings. The annual cost of semaglutide would need to be reduced 3-fold, from $13 618 to $3591, for it to be a cost-competitive alternative.

**Meaning:**

The study suggests that while semaglutide is effective for weight loss, it is not economically viable over the long term compared with ESG, which remains a cost-saving alternative for this patient population.

## Teaser

This economic evaluaton study assesses the cost-effectiveness of semaglutide compared with endoscopic sleeve gastroplasty over 5 years for individuals with class II obesity.

## Introduction

Obesity is considered a global pandemic. In the US, the prevalence of obesity was 42.4% in 2018 and is estimated to be approximately 50% by 2030.^1,2^ It is associated with chronic medical conditions that affect morbidity and mortality, causing a significant burden on annual US health care spending. In 2016, the direct medical costs of obesity were estimated to be $260 billion.^3^ Given the high prevalence of obesity in the US, with its adverse consequences on health and health care economics, it is important to understand the cost-effectiveness of available interventions.

Lifestyle intervention and bariatric surgery represent 2 extreme ends of the spectrum of obesity treatment. Lifestyle interventions have limitations due to the burden of lifestyle changes and poor efficacy, while bariatric surgery remains underused due to its perceived invasiveness, cost, and limited insurance coverage.^4,5,6^ Alternatively, endoscopic bariatric and metabolic therapies have evolved as an effective, safe, and minimally invasive option for the treatment of obesity.^7,8,9,10^ Endoscopic sleeve gastroplasty (ESG) is the most effective endoscopic bariatric and metabolic therapy that is attracting attention worldwide.^7,11,12,13^ It is an incisionless, per-oral, minimally invasive endoscopic procedure that applies full-thickness sutures along the greater curvature of the stomach, from the inside, to reduce gastric capacity and alter gastric motility.^14^ In addition, new weight-loss medications, such as glucagon-like peptide-1 receptor agonists (eg, semaglutide and liraglutide), have recently attracted increased attention. They have several proposed mechanisms of action, including a delay in gastric emptying, which results in an increased duration of satiety and decreased appetite. In particular, semaglutide has gained popular appeal due to its noninvasiveness, ease of use as a weekly injection, and short-term effectiveness.^15^ However, their widespread use could strain the budget of most payers, including Medicare.^16^ Although glucagon-like peptide-1 receptor agonists and ESG are seeing markedly increased use, their comparative cost-effectiveness is unknown.

In the Multicenter ESG Randomized Interventional Trial (MERIT), ESG plus lifestyle adjustments achieved a 49.2% excess weight loss at 1 year compared with 3.2% in the control group.^17^ On the other hand, the Semaglutide Treatment Effect in People With Obesity (STEP 1) study showed that semaglutide with lifestyle changes resulted in a 14.9% total body weight reduction over 68 weeks, a significant improvement compared with the 2.4% total body weight reduction among the placebo group, with 86.4% of semaglutide recipients losing over 5% of their body weight.^15^ Hence, semaglutide and ESG have established effectiveness and safety profiles from randomized clinical trials (STEP 1 and MERIT).^15,17^ Therefore, our economic evaluation study aimed to perform a cost-effectiveness analysis of semaglutide and ESG among patients with obesity from a US health care perspective.

## Methods

### Model Overview

The base case was a 45-year-old patient with class II obesity (body mass index [BMI] of 35-39.9 [calculated as weight in kilograms divided by height in meters squared]) and a BMI of 37. For this economic evaluation study conducted from September 1, 2022, to May 31, 2023, we developed a state-transition Markov cohort model to assess the cost-effectiveness of 2 strategies: semaglutide and ESG. The model time horizon was 5 years to capture the outcomes of interest. The first-year clinical data were derived from 2 randomized clinical trials (STEP 1 [semaglutide] and MERIT [ESG]).^15,17^ The data for the following years (years 2-5) were derived from published studies and publicly available data sources (Table 1).^12,15,17,18,19,20,21,22,23,24,25,26,27^ The state-transition time or model cycle length was 1 month. eFigure 1A and B in Supplement 1 shows a simplified version of the simulated strategies in the model. The study did not require institutional review board approval because it used publicly available data to simulate hypothetical patients. The study was reported in alignment with the Consolidated Health Economic Evaluation Reporting Standards (CHEERS) reporting guideline for economic analyses.^28^

**Table 1.** Base-Case Model Inputs

Parameter	Base case (range)	Comments	Distribution	Source
Treatment-related probabilities or outcomes				
Monthly change in BMI with ESG (year 1)	−0.40 (−0.36 to −0.44)	±10%	Normal	Abu Dayyeh et al,^17^ 2022 (MERIT)
Monthly change in BMI with ESG (years 2-5)	0.0065 (0.0059 to 0.0072)	±10%	Normal	Hedjoudje et al,^12^ 2020; Sharaiha et al,^18^ 2021
Monthly change in BMI with semaglutide (year 1)	−0.34 (−0.31 to −0.38)	±10%	Normal	Wilding et al,^15^ 2021 (STEP 1 trial)
Monthly change in BMI with semaglutide (years 2-5)	0.0012 (0.0013 to 0.0011)	±10%	Normal	Marso et al,^19^ 2016
Monthly change in BMI for dropouts	0.138 (0.124 to 0.152)	±10%	Normal	Smith et al,^20^ 2010
Monthly change in BMI with no semaglutide or ESG	0.0127 (0.0114 to 0.0140)	±10%	Normal	Malhotra et al,^21^ 2013
Annual dropout rate (year 1) with semaglutide, %	10 (6 to 14)	Trial data	Beta	Wilding et al,^15^ 2021 (STEP 1 trial); Garvey et al,^22^ 2022 (STEP 5 trial); O’Neil et al,^23^ 2018
Annual dropout rate (years 2-5) with semaglutide, %	3.25 (1.95 to 4.55)	Trial data	Beta	O’Neil et al,^23^ 2018; Marso et al,^19^ 2016
30-d Mortality with ESG, %	0.2 (0.1 to 0.3)	NA	Beta	Expert opinion
Annual rate of minor complications with ESG, %	10 (6 to 14)	±40%	Beta	Abu Dayyeh et al,^17^ 2022 (MERIT)
Annual rate of major complications with ESG, %	2 (1 to 3)	NA	Beta	Abu Dayyeh et al,^17^ 2022 (MERIT)
Repeat procedure, ESG, %	16 (13 to 19)	±20%	Beta	Expert opinion
Quality-of-life estimates				
Class II obesity (aged 41-50 y)	0.79 (-)	NA	Beta	Alsumali et al,^24^ 2018
Initial surgery	−0.22 (−0.24 to −0.20)	1 wk Applied to ESG	Beta	Campbell et al,^25^ 2010
Minor complications	−0.11 (−0.12 to −0.10)	2 wk Applied to ESG	Beta	Campbell et al,^25^ 2010
Major complications	−0.36 (−0.40 to −0.32)	2 wk Applied to ESG	Beta	Campbell et al,^25^ 2010
Improvement per 1-unit decrease in BMI	0.0056 (0 to 0.017)	NA	Beta	Klebanoff et al,^26^ 2017
Costs				
Initial surgery, ESG (2022), $	16 360 (12 270 to 20 450)	±25%	Gamma	Institutional data
Annual cost, semaglutide (2022), $	13 618 (10 214 to 17 023)	±25%	Gamma	ICER report,^27^ 2022
Major complications with ESG (2022), $	32 840 (24 630 to 41 050)	±25%	Gamma	Campbell et al,^25^ 2010
Minor complications with ESG (2022), $	2676 (2007 to 3346)	±25%	Gamma	Campbell et al,^25^ 2010

Abbreviations: BMI, body mass index; ESG, endoscopic sleeve gastroplasty; ICER, Institute for Clinical and Economic Review; MERIT, Multicenter ESG Randomized Interventional Trial; NA, not applicable; STEP, Semaglutide Treatment Effect in People With Obesity.

### Competing Strategies for Management of Class II Obesity

We simulated 2 treatment strategies vs no treatment: semaglutide and ESG. Our reference group included patients who did not undergo any treatment for weight loss and had a slight weight gain over time based on published literature.^21^ Patients in the semaglutide group faced the risk of dropping out of the strategy due to medication intolerance.^15,19,22,23^ Based on published literature, patients who dropped out of the weight loss strategy regained weight.^20,29^ Patients in the ESG group faced the risk of severe adverse events (defined as class 3-5 on the Clavien-Dindo classification scale,^30^ requiring surgical, endoscopic, or radiologic intervention) and adverse events (defined as class 1-2 on the Clavien-Dindo classification scale, including accommodative gastrointestinal symptoms), which were based on data from the MERIT trial.^17^ Adverse event rates for the ESG strategy were applied for the first year after the procedure. Patients who did not have a satisfactory response underwent repeat ESG. For model input, weight change was converted to the rate of BMI change.^26,29,31^ The background mortality table for the year 2020 in the US was adjusted for age, sex, and BMI using data from the US Third National Health and Nutrition Examination Survey.^32^

### Costs and Quality-of-Life Adjustments

The model costs assume a US health care system’s perspective. The cost of no treatment was assumed to be zero. The model incorporated costs of ESG, repeat ESG, associated adverse events from ESG, and the monthly cost of semaglutide.^25,27^ All costs from prior years were adjusted to 2022 US dollars using the Consumer Price Index.^33^ Patients in the ESG group received an initial quality-of-life decrement associated with the procedure, which was applied for 1 week. The association of adverse events from ESG with quality of life was also incorporated; a quality-of-life decrement was applied for 2 weeks each for severe adverse events and adverse events.^24,25^ We did not apply any quality-of-life decrement for semaglutide intolerance or adverse events because this typically involves stopping the medication and recurrence of weight gain, which is already captured in the model. We did not apply any cost associated with obesity because the model schematic did not project resolution of obesity; however, we instead incorporated improvement in quality of life with weight loss. We applied a quality-of-life improvement of 0.0056 quality-adjusted life-years (QALYs) per BMI unit decrease, as used previously.^26,29,31^ Costs and utilities were discounted at an annual rate of 3%.^34^

### Outcomes

Our outcomes of interest were QALYs, total costs, and incremental cost-effectiveness ratios (ICERs), which were calculated as differences in costs and QALYs between the competing strategies. A willingness-to-pay threshold of $100 000 per QALY was used to evaluate cost-effectiveness.

### Statistical Analysis

To ensure the robustness and reliability of our model’s outcomes, we conducted a comprehensive sensitivity analysis, including 1-way, 2-way, and probabilistic sensitivity analyses. Deterministic sensitivity analyses were performed by varying one parameter at a time within prescribed bounds and recording the change in ICERs. Probabilistic sensitivity analysis is a technique that determines the association of uncertainty in various model inputs with the estimated results, addressing the variability and probabilistic nature of the model parameters. We selected specific probability distributions for each parameter based on their statistical properties and relevance to the data type (eTable in Supplement 1). Probabilistic sensitivity analyses were performed by sampling all parameters simultaneously from probability distributions. Gamma distributions were used for costs, normal distributions for weight change, and beta distributions for all other parameters. Probabilistic sensitivity analysis was performed on the model on various time horizons. In addition, we used second-order Monte Carlo simulations to incorporate parameter uncertainty into the model, running 10 000 iterations of the model, each time drawing a different set of parameters from their respective distributions. The percentage of times each strategy was cost-effective at the willingness-to-pay threshold was recorded.

We assumed that patients who dropped out from the semaglutide strategy experienced weight loss for at least 3 months before starting to regain weight. For the ESG strategy, a proportion of patients with insufficient weight loss or weight regain underwent repeat ESG after the first year. Although MERIT or other published literature did not report any mortality associated with ESG, patients faced a 30-day mortality risk in our model based on expert opinion. We made this assumption based on the procedure’s invasiveness compared with semaglutide. The analysis was conducted using TreeAge Pro, version 2023 R2 (TreeAge Software).

## Results

In the base-case analysis, the QALYs accumulated over 5 years were 3.55 for no treatment, 3.60 for semaglutide, and 3.66 for ESG (Table 2). The semaglutide strategy cost $33 583 more than the ESG strategy over this time horizon. Endoscopic sleeve gastroplasty was cost saving, with lower cost and higher QALYs. Due to medication intolerance or other causes, approximately 20% of modeled patients dropped out of the semaglutide strategy. Endoscopic sleeve gastroplasty achieved and sustained greater weight loss compared with semaglutide over a 5-year time horizon for the modeled patients (BMI of 31.7 vs 33.0).

**Table 2.** Base-Case Results Over Different Time Horizons

No. of months	Strategy	Costs, $		QALYs		ICER ($/QALY)	NMB, $	BMI
		Cumulative	Incremental	Cumulative	Incremental			
60	No semaglutide or ESG	NA	NA	3.55	NA	NA	NA	37.8
12	ESG	17 229	5488	0.72	0.002	240 265	54 996	32.2
12	Semaglutide	11 742		0.72		0	60 255	32.9
24	ESG	19 685	3162	1.47	−0.009	0	127 288	32.2
24	Semaglutide	22 848		1.46		−347 584	123 216	32.9
36	ESG	19 685	14 003	2.23	−0.02	0	202 853	31.6
36	Semaglutide	33 688		2.20		−599 580	186 515	32.9
48	ESG	19 685	24 129	2.95	−0.04	0	275 691	31.7
48	Semaglutide	43 814		2.92		−617 831	247 653	32.9
60	ESG	19 685	33 583	3.66	−0.06	0	345 854	31.7
60	Semaglutide	53 268		3.60		−595 532	306 632	33.0

Abbreviations: BMI, body mass index (calculated as weight in kilograms divided by height in meters squared); ESG, endoscopic sleeve gastroplasty; ICER, incremental cost-effectiveness ratio; NA, not applicable; NMB, net monetary benefit; QALY, quality-adjusted life-year.

Over 1 year, ESG was not cost-effective compared with semaglutide, with an ICER of $240 265/QALY (Table 2). However, when the time horizon was extended to 2 years, ESG became cost saving and dominated the semaglutide strategy.

**Figure 1.**
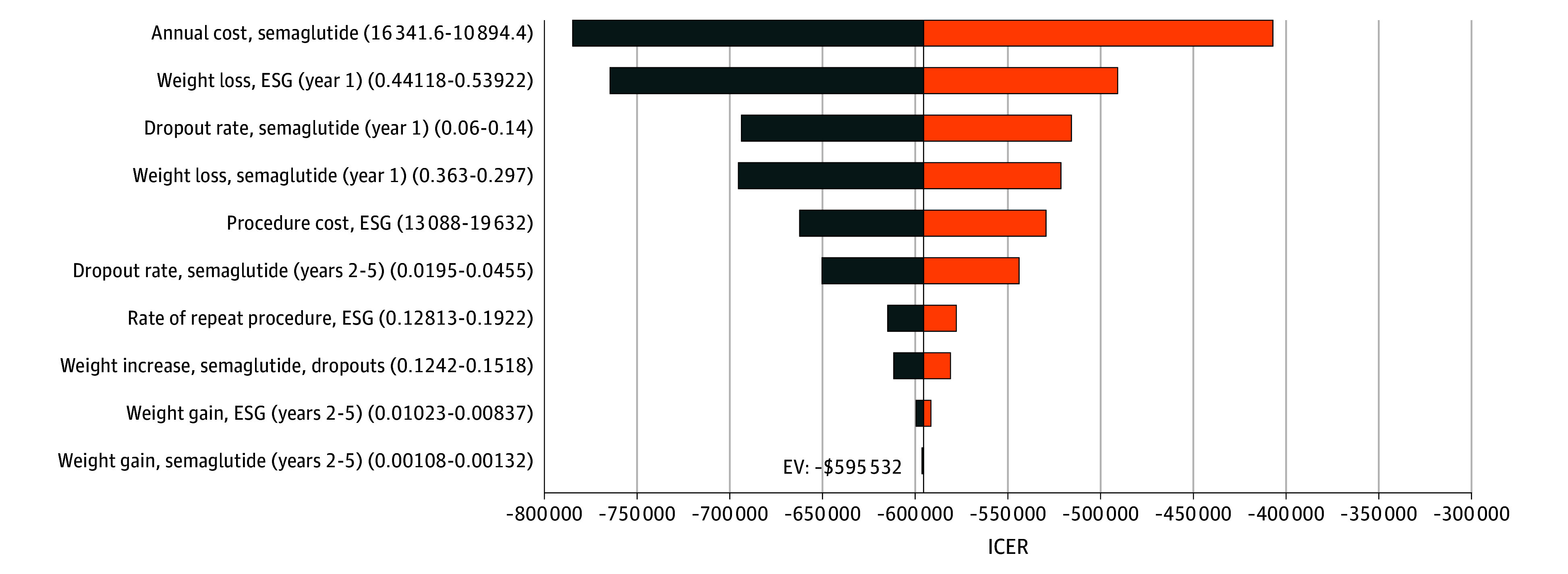
Results of 1-Way Sensitivity Analyses Performed Over a 5-Year Time Horizon One-way sensitivity analysis involves adjusting the value of 1 model parameter at a time to assess the association with study outcomes. This figure includes the 10 parameters with the largest association with incremental cost-effectiveness ratio (ICER) values when modified. The numbers on either side of the bars indicate the extreme parameter values associated with the resulting ICER shown in the figure. This figure is centered around the base case with an ICER of –$595 532 per quality-adjusted life-year. ESG indicates endoscopic sleeve gastroplasty; EV, expected value.

**Figure 2.**
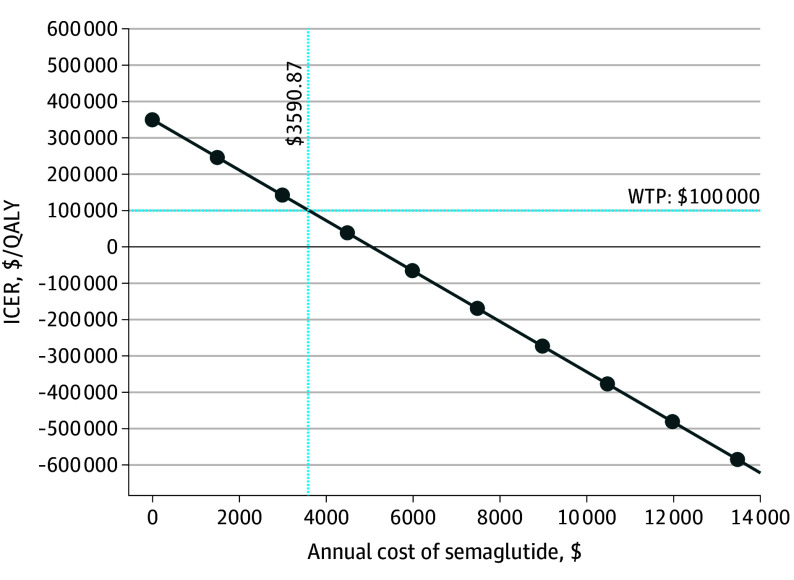
Cost-Threshold Analysis of Semaglutide Compared With Incremental Cost-Effectiveness Ratio (ICER) of Endoscopic Sleeve Gastroplasty Over 5-Year Time Horizon, With Willingness-to-Pay (WTP) Threshold of $100 000 per Quality-Adjusted Life-Year (QALY) The cost threshold at which semaglutide can be nondominant to endoscopic sleeve gastroplasty at the 5-year time horizon is $3591.

We performed sensitivity analyses over a 5-year time horizon, with ESG remaining cost saving in all analyses, with an ICER of –$595 532 per QALY for the base case (Figure 1). Probabilistic sensitivity analysis was performed on the model over varied time horizons. With the use of a willingness-to-pay threshold of $100 000 per QALY, ESG was cost-effective compared with semaglutide, with a probability of 1.00%, 80.90%, 99.97%, 100%, and 100% over 1, 2, 3, 4, and 5 years, respectively (Table 3). At a 1-year time horizon, the cost required for the ESG procedure to become cost-effective compared with semaglutide with an ICER threshold of $100 000/QALY, currently $16 360, was $11 098 (eFigure 2 in Supplement 1). The annual price of semaglutide to achieve nondominance compared with ESG with an ICER threshold of $100 000/QALY over a 5-year time horizon was $3591 and is currently at $13 618 (Figure 2). Two-way sensitivity analyses of the annual cost of semaglutide to clinically relevant parameters demonstrated that the choice of strategy would only change if the cost of semaglutide decreased by at least 3-fold (eFigure 3A-D in Supplement 1).

**Table 3.** Probabilistic Sensitivity Analysis Over Varied Time Horizons^a^

Strategy	Probability treatment is cost-effective, %				
	At year 1	At year 2	At year 3	At year 4	At year 5
ESG	1.00	80.90	99.97	100.0	100.0
Semaglutide	99.00	19.10	0.03	0.0	0.0

^a^ The model was run using second-order sampling for 100 000 iterations for each time horizon; the percentage of these times in which that ESG was cost-effective compared with semaglutide is shown using a willingness-to-pay threshold of $100 000/QALYs.

## Discussion

Our economic evalution demonstrates that ESG achieved and sustained greater weight loss over a 5-year time horizon compared with semaglutide (BMI of 31.7 vs 33.0). Furthermore, ESG is cost saving compared with semaglutide for patients with class II obesity. These results remained robust in sensitivity analyses. The strategic choice of cost-saving yet effective treatment such as ESG compared with semaglutide for specific patient groups could help alleviate the potential budget strain expected from the use of semaglutide.^16^ Given the high prevalence of obesity in the US, there is a growing need for cost-effective interventions that can be made accessible to the broader population—to those without the ability to pay out of pocket or from limited use of effective interventions because of budget constraints—to address the obesity pandemic.

Although few studies have assessed the cost-effectiveness of semaglutide,^27,29,31^ our study is unique in comparing it with a widely performed, minimally invasive, incisionless endoscopic procedure (ie, ESG). The data from MERIT and the STEP 1 trial provide high-level evidence for ESG and semaglutide, respectively.^15,17^ A strength of our analyses lies in using first-year data from these randomized clinical trials with the following years’ data from published literature to assess the cost-effectiveness of the 2 strategies over a 5-year time horizon. This provides a reasonable time frame for comprehensively assessing the cost-effectiveness of the 2 strategies, which has important implications for patients, health care professionals, and policymakers for medical decision-making at individual and population levels.

In all 1-way sensitivity analyses over a 5-year time horizon, ESG remained cost saving, which included varying the probability of procedural mortality, quality-of-life decrements with the procedure, or associated severe adverse events and adverse events. To achieve nondominance compared with ESG, the annual price of semaglutide, currently $13 618, must be $3591. This finding was further varied on 2-way sensitivity analyses with weight loss after ESG, rate of repeat ESG, weight loss with semaglutide, and dropout rate with semaglutide. It was concluded that for all parameters, the cost of semaglutide must be decreased by at least 3-fold to cause any change in the strategy preference. The Institute for Clinical and Economic Review found that the price of semaglutide must be lowered to $7494 to be cost-effective compared with lifestyle modification.^27^ This finding means the higher the effectiveness of the comparative strategy, the lower the cost of semaglutide required to achieve commonly accepted benchmarks for cost-effectiveness. These findings are significant for both health care professionals and patients with obesity because they compare the risks and benefits associated with a noninvasive medication against those of an emerging minimally invasive endoscopic option.

Through mathematical modeling of published literature, our study calculated that approximately 20% of patients would drop out of the semaglutide group over 5 years due to intolerance or other causes. The dropout rate due to inability to tolerate adverse events or other reasons was 7% in the STEP 1 trial and 13.5% in the STEP 8 trial over 68 weeks.^15,35^ This rate does not include patients who could not achieve maximal dosage. As such, we believe our estimate of a 20% dropout rate at 5 years is conservative and that the dropout rate could be higher considering continued use of the medication to maintain weight loss with costs accruing over time.^36^

### Limitations and Strengths

Our study has some limitations. In our analysis, we did not explicitly model the benefit associated with improvement in comorbidities, such as hypertension, hyperlipidemia, or type 2 diabetes, from either strategy. However, we accounted for this association by incorporating BMI-specific mortality, an approach that has been previously used.^26^ In addition, the STEP 1 trial excluded all patients with diabetes, whereas MERIT conditionally included patients with type 2 diabetes.^15,17^ Therefore, we adopted an approach not to include improvement in comorbidities separately in the model. Furthermore, our study incorporated relatively short-term data for both strategies because long-term data are still accumulating. However, we did not want to make assumptions or impute data for our model and instead relied on published literature, limiting the time horizon. Our model did not account for the microlevel follow-up costs, such as routine clinic visits, assuming that these costs would be comparable for both treatment arms. Such costs are unlikely to have a significant association with the overall cost-effectiveness results, a perspective consistent with established practices in the existing literature on health care interventions.^26,37^ Last, we acknowledge that as more obesity medications are approved, market trends may moderately lower the price of semaglutide. However, the specialized manufacturing of peptide-based drugs implies that significant cost reductions are unlikely.

Despite these limitations, our study has several strengths. We compared ESG with semaglutide, specifically among patients with class II obesity, because it is clinically relevant and reflective of common treatment practices.^38^ A recent study by Saumoy et al^39^ strengthens our results. Still, our study is unique because it relies exclusively on observed outcomes with minimal assumptions, enhancing the accuracy and reliability of our cost-effectiveness estimates for these treatments. Last, we ran our model over a 5-year time horizon to not only incorporate best available evidence for both strategies but also to capture health outcomes and costs comprehensively. The longer time horizon would make ESG even more cost saving.

## Conclusions

This economic evalution study suggests that ESG is cost saving compared with semaglutide for class II obesity. This finding is due to the increased effectiveness and lower costs of ESG and the increased dropout rates over time with semaglutide. The annual price of semaglutide must decrease by more than 3-fold to achieve nondominance with ESG.

## Notes

Supplement 1. **eFigure 1.** Simplified State Transition Diagram for Semaglutide and Endoscopic Sleeve Gastroplasty **eFigure 2.** Cost-Threshold Analysis of ESG Compared to ICER of Semaglutide Over 1 Year Time Horizon, With Willingness-to-Pay Threshold of $100,000/QALY **eFigure 3.** Two-Way Sensitivity Analysis **eTable.** Variable Parameters With Rationale of Their Chosen Distributions

Supplement 2.
Data Sharing Statement
