# Supplementary material for: Semaglutide vs Endoscopic Sleeve Gastroplasty for Weight Loss
Source: JAMA Netw Open. 2024 Apr 12;7(4):e246221. doi: 10.1001/jamanetworkopen.2024.6221 (PMC11015347; doi:10.1001/jamanetworkopen.2024.6221)
Supplement: Supplement 1. — eFigure 1. Simplified State Transition Diagram for Semaglutide and Endoscopic Sleeve Gastroplasty eFigure 2. Cost-Threshold Analysis of ESG Compared to ICER of Semaglutide Over 1 Year Time Horizon, With Willingness-to-Pay Threshold of $100,000/QALY eFigure 3. Two-Way Sensitivity Analysis eTable. Variable Parameters With Rationale of Their Chosen Distributions [file jamanetwopen-e246221-s001.pdf]

Supplementary Online Content

Haseeb M, Chhatwal J, Xiao J, Jirapinyo P, Thompson CC. Semaglutide vs endoscopic sleeve gastroplasty for weight loss. *JAMA Netw Open.* 2024;7(4):e246221. doi:10.1001/jamanetworkopen.2024.6221

eFigure 1. Simplified State Transition Diagrams for Semaglutide and Endoscopic Sleeve Gastroplasty
eFigure 2. Cost-Threshold Analysis of ESG Compared to ICER of Semaglutide Over 1 Year Time Horizon, With Willingness-to-Pay Threshold of $100,000/QALY
eFigure 3. Two-Way Sensitivity Analysis
eTable. Variable Parameters With Rationale of Their Chosen Distributions

This supplementary material has been provided by the authors to give readers additional information about their work.

© 2024 Haseeb M et al. *JAMA Network Open.*

eFigure 1. Simplified State Transition Diagrams for Semaglutide and Endoscopic Sleeve Gastroplasty

eFigure 1A. Simplified State Transition Diagram for Semaglutide. QALY, quality-adjusted life years; ICER, incremental cost-effectiveness ratio; BMI, body mass index.

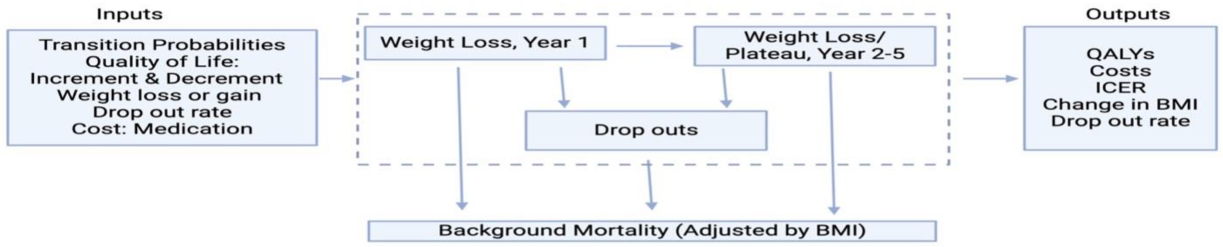

© 2024 Haseeb M et al. *JAMA Network Open.*

eFigure 1B. Simplified State Transition Diagram for Endoscopic Sleeve Gastroplasty. The repeat procedural rate was applied to the model in year 2 for model simplicity. All patients modeled to undergo repeat procedure were subjected to the same risks and costs as the initial procedure, which was also factored in the same year. QALY, quality-adjusted life years; ICER, incremental cost-effectiveness ratio; BMI, body mass index.

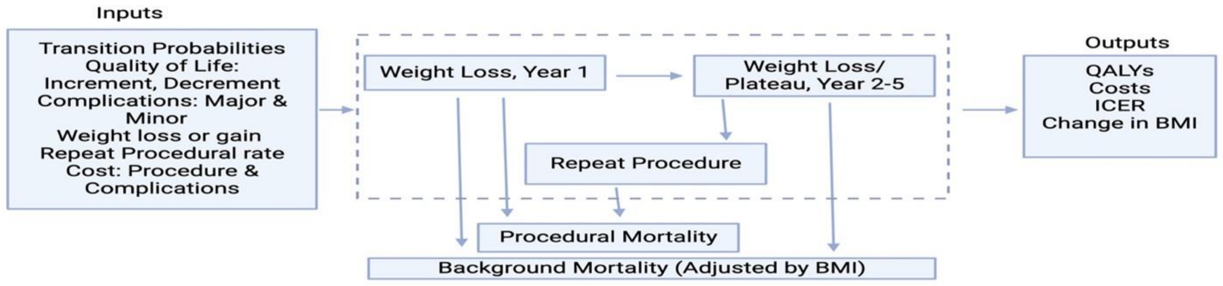

© 2024 Haseeb M et al. *JAMA Network Open.*

eFigure 2. Cost-Threshold Analysis of ESG Compared to ICER of Semaglutide Over 1 Year Time Horizon, With Willingness-to-Pay Threshold of $100,000/QALY
It shows a cost-threshold of $11,098 at which ESG will be cost-effective compared to semaglutide at 1 year time horizon.

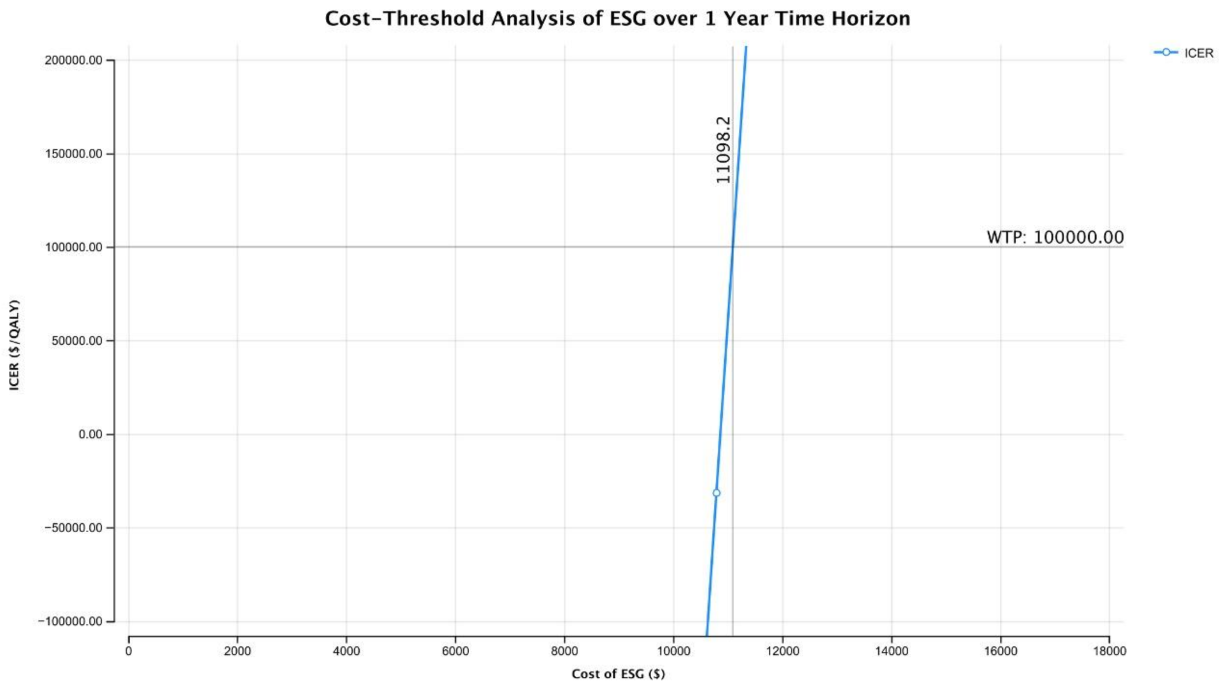

© 2024 Haseeb M et al. *JAMA Network Open.*

eFigure 3. Two-Way Sensitivity Analysis
The impact of the annual cost of semaglutide on two-way sensitivity analyses with the following clinically relevant parameters: A: Weight loss with semaglutide, B: Dropout rate with semaglutide, C: Weight loss after ESG, D: Rate of repeat ESG. The results indicate that a change in strategy preference would only occur if the cost of semaglutide is decreased by at least three-fold.

3A:

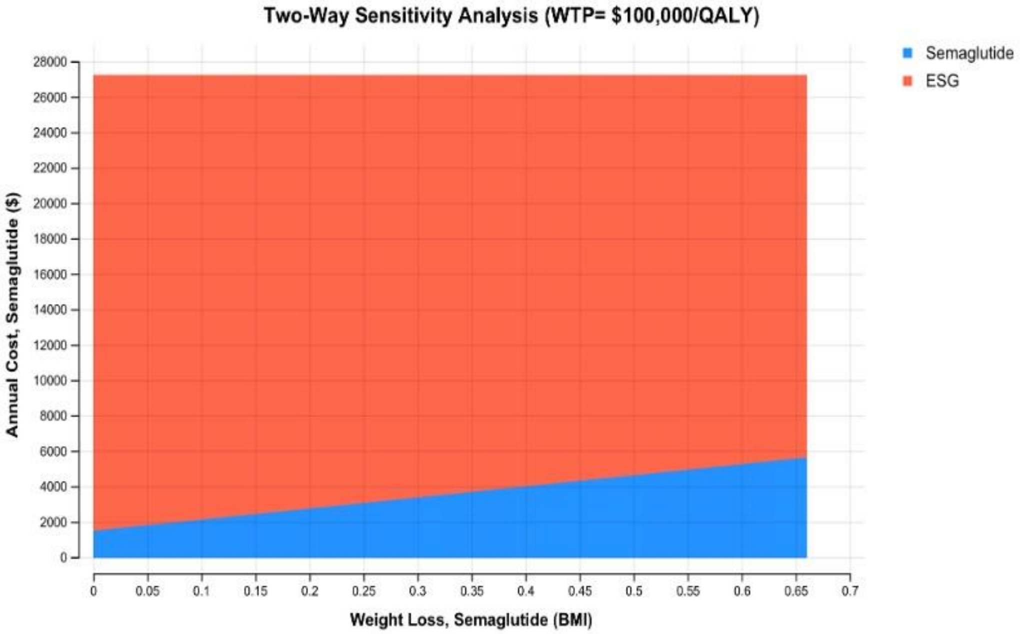

© 2024 Haseeb M et al. *JAMA Network Open.*

3B:

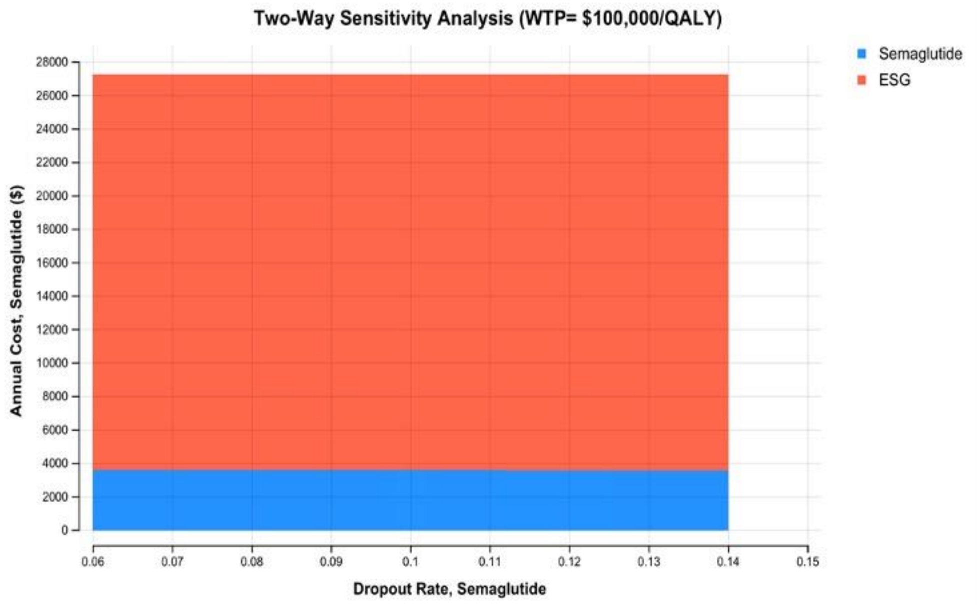

© 2024 Haseeb M et al. *JAMA Network Open.*

3C:

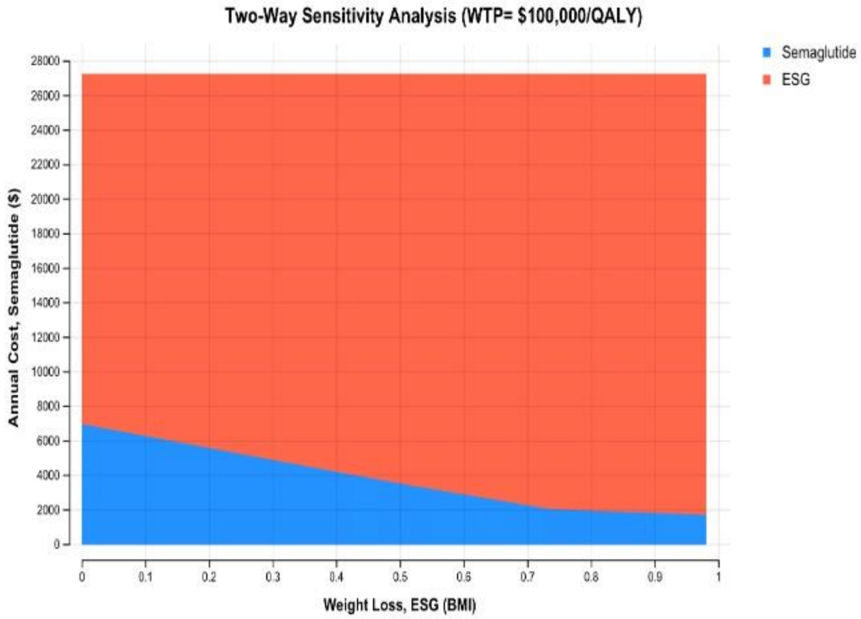

© 2024 Haseeb M et al. *JAMA Network Open.*

3D:

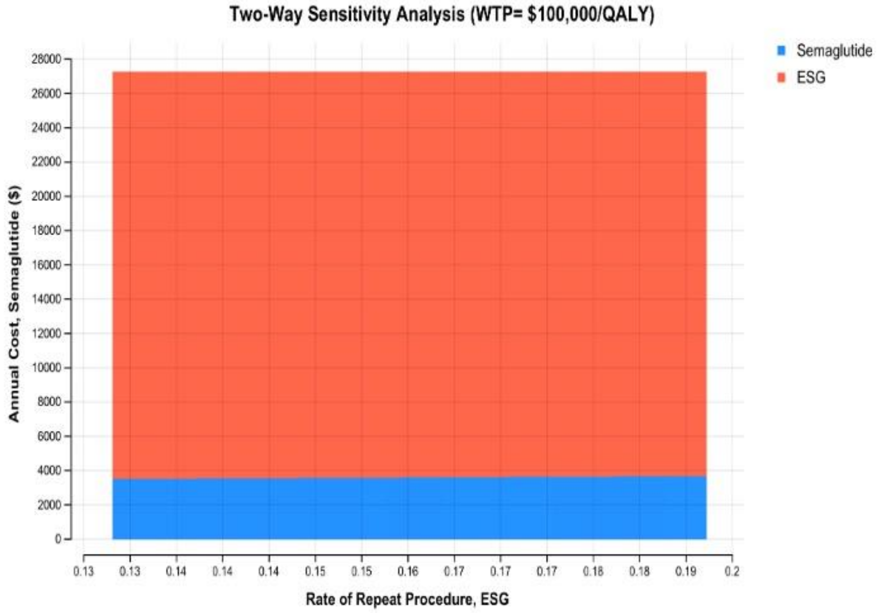

© 2024 Haseeb M et al. *JAMA Network Open.*

eTable. Variable Parameters With Rationale of Their Chosen Distributions

Parameter Type	Distribution Chosen	Rationale
Cost Parameters	Gamma Distribution	Suitable for skewed data, allowing for the accommodation of the long tail of high-cost events typical in healthcare expenditure.
Transition Probabilities	Beta Distribution	Constrained between 0 and 1, matching the nature of probabilities and rates, ideal for parameters such as treatment effectiveness and adverse event rates.
Utility Values	Beta Distribution	Reflects the bounded nature of utility values which are also constrained between 0 and 1, suitable for quality-adjusted life-year (QALY) estimations.
Normal Distribution parameters (e.g. age, weight)	Normal Distribution	Appropriate for parameters that are symmetrically distributed around a mean, often used for biological or human-related continuous variables.

© 2024 Haseeb M et al. *JAMA Network Open.*
