# Supplementary material for: Semaglutide vs Endoscopic Sleeve Gastroplasty for Weight Loss
Source: JAMA Netw Open. 2024 Apr 12;7(4):e246221. doi: 10.1001/jamanetworkopen.2024.6221 (PMC11015347; doi:10.1001/jamanetworkopen.2024.6221)
Supplement: Supplement 2. — Data Sharing Statement [file jamanetwopen-e246221-s002.pdf]

Data Sharing Statement

Haseeb. Semaglutide vs Endoscopic Sleeve Gastroplasty for Weight Loss. *JAMA Netw Open*. Published April 12, 2024. doi:10.1001/jamanetworkopen.2024.6221

Data
Data available: No

Additional Information
Explanation for why data not available: Data will be made available on a case by case basis with a data sharing agreement made between Brigham and Women's Hospital and requesting institution.
